# Finite Element Analysis of Steel Plates with Rectangular Openings Subjected to Axial Stress

**DOI:** 10.3390/ma15134421

**Published:** 2022-06-22

**Authors:** Ahmad Mahamad Al-Yacouby, Arwie Amri Mazli, M. S. Liew, R. M. Chandima Ratnayake, Samindi M. K. Samarakoon

**Affiliations:** 1 Civil and Environmental Engineering Department, Universiti Teknologi PETRONAS, Seri Iskandar 32610, Perak, Malaysia; arwie_17000223@utp.edu.my (A.A.M.); shahir_liew@utp.edu.my (M.S.L.); 2 Department of Mechanical and Structural Engineering and Materials Science, University of Stavanger, 4021 Stavanger, Norway; chandima.ratnayake@uis.no (R.M.C.R.); samindi.samarakoon@uis.no (S.M.K.S.)

**Keywords:** steel plates, rectangular openings, stress analysis, finite element analysis

## Abstract

Steel plates with openings are among the important ship structural components used in the ship’s hull to withstand the hydrostatic forces of the ocean, which cause sagging and hogging moments at the ship’s bottom. The existence of openings on plates can cause structural rupture, stress concentration and a decrease in ultimate strength. This research is aimed at investigating the influence of selected parameters on the ultimate capacity of steel plates with rectangular holes subjected to axial stress, using ANSYS finite element analysis (FEA) under its non-linear static structural programme. The main parameters investigated in this paper are the plate thickness, opening aspect ratio, number of openings, position of openings, and the boundary condition of the plate. The influence of these parameters on the stress of plates and their deformation was evaluated. The comparison of the numerical simulation with the well-established analytical method using the Navier solution and Roark’s Formulas showed a good agreement.

## 1. Introduction

### 1.1. Literature Review

**Figure 1:**
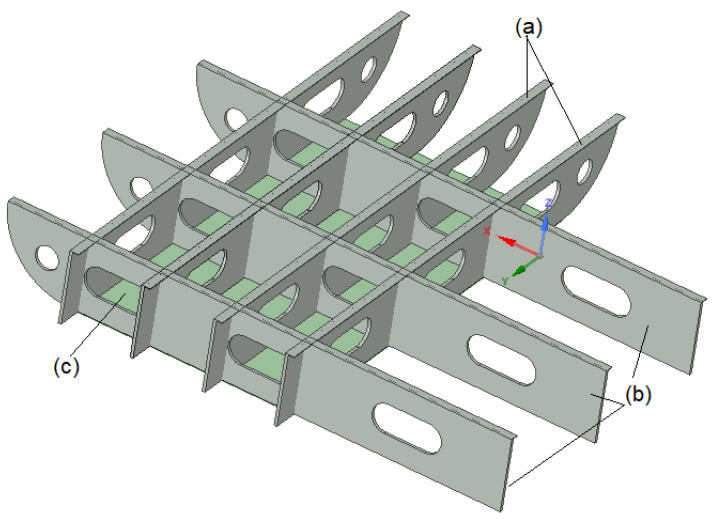
Plate floor details used in shipbuilding industry: (a) Flanges, (b) Traverse plate floor, (c) Cardboard strake.

In the shipbuilding industry, steel plates with openings, usually known as floor plates, as shown in Figure 1, are commonly used in the building of a ship’s hull. The openings in the steel floor plates are needed for different purposes, including for inspection of the hull, to place HVAC (heating, ventilation and air conditioning) systems and pipes/cables and for other design reasons [1]. The hull of a ship is a box girder structure composed of plates and stiffeners. The main loads acting on a hull girder are distributed lateral loads, such as hull weight and cargo weight, as well as buoyancy force and wave force [2]. The floor plates are important ship structural components because they resist the bending stresses on the ship’s hull when the ship undergoes sagging and hogging due to the hydrostatic and hydrodynamic loads. The floor plates and other ship’s hull components are aligned together longitudinally along the length of the ship. The distributed loads in the vertical direction may produce bending deformation in a hull girder. Under sagging conditions, the deck plate is subjected to thrust and the bottom plate to tension [2]. Failure of the floor plates at the ship hull can be due to shear, bending and buckling from the sagging and hogging of the ship, and the weight of the equipment and cargo of the ship. An example of a ship hull’s plating failure is the MOL Comfort container ship, which split in half and sank [3]. Moreover, openings in the hull of a ship normally introduce a discontinuity that can result in fatigue damage due to cyclic loading [4,5].

The review of the literature shows that several research work on steel plates have been published, with the pioneering studies started in the 1930s. The first pioneering rework on the ultimate strength of perforated plates under uniaxial compression loads was carried out by Vann [6]; The assessment of the ultimate strength of steel plates under uniaxial compression is a basic practice to ensure the safe design of ship structures [7,8,9]. The majority of these studies address floor plates with either circular or elliptical openings. In the last decade, computer-based engineering simulation incorporated with FEA has become a research tool used for the assessment and appraisal of modelled complex engineering structures. Moreover, not only the use of circular or elliptical openings, but also the potential use of rectangular openings in floor plates have been investigated using FEA and experimental campaigns [1,10,11].

Zhao et al. [10] investigated the shear capacity of perforated steel plates against the effects of varying parameters—the opening’s shape and position and the plate’s thickness and material strength—through analytical, laboratory and simulation approaches. On the effect of the opening’s shape, it was found that plates with rectangular openings have higher shear capacity than plates with circular holes [10]. However, steel plates with rectangular openings experienced a failure mode which could be detrimental to the steel plates.. Besides this, a study by Yu and Lee [11] on the effects of opening’s aspect ratio on the ultimate strength capacity of perforated steel plates subjected to uniaxial compression reported that the plate with transverse holes had higher strength capacity than the plate with longitudinal holes. Yu and Lee [11] also established a new design expressions for the ultimate strength of steel plates with rectangular holes using empirical approach. As for the effect of length-to-thickness ratio of functionally graded plates, an increase in the length-to-thickness ratio resulted in reduction in the deflection of the plates. Therefore, based on the aforementioned findings, it can be concluded that rectangular openings in floor plates show promising results and further research verifications are necessary.

### 1.2. Importance of the Research

The presence of openings on steel plates represents a structural rupture that affects the stress distribution and the ultimate strength of steel plates [1]. Openings in steel plates are needed to serve as access points for engineering services and systems in building, marine and offshore structures industry. The application of reinforcements on perforated plates is useful techniques to compensate the strength loss of steel plates with openings. Stiffened steel plates are plates with beams welded longitudinally, transversely, or both, on one face of the steel plate. According to Lima et al. [12], who studied the effect of stiffener configuration on the ultimate buckling stress of steel plates, reported that regardless of the configuration of stiffeners, stiffened plates have a better ultimate buckling stress as compared to a standalone steel plate. Structural elements in the form of plates and shells are encountered in many engineering applications, such as civil, mechanical, aeronautical, marine and chemical engineering. When suitably designed, even very thin plates, and especially shells, can support large loads [13,14]. For additional references on the topic, the reader can refer to [10,15]. However, despite the importance of steel plates with openings in engineering applications, there are limited studies on the influence of different opening configurations, number of openings, plate thickness and aspect ratio on the ultimate strength and deformations in steel plates with rectangular holes.

The primary objective of this manuscript is to perform stress analysis on steel plates with rectangular openings exposed to axial loads, using FEA for different opening configurations, number of openings, boundary conditions, plate thicknesses and aspect ratio. The steel plates with openings are modelled using non-linear static finite element analysis using ANSYS software. The results obtained from the ANSYS simulation of the stress and displacement of steel plates without openings were validated with the well-known Navier solution and Roark’s Formulas. The parameters investigated in this manuscript are the steel plate’s thickness, opening ratio, opening number, opening position and the boundary conditions. The outline of the remaining sections of this article is as follows. Section 2 provides the theoretical background of the analytical approach; Section 3 introduces the materials and methods adopted in this study; Section 4 presents the results and discussion, and the conclusion is presented in Section 5.

## 2. Theoretical Background

### Analytical Method of Determining Stress and Displacement of Unperforated Steel Plate

Practically, to determine the stress and displacement of steel plate without openings, the Navier solution is commonly used. The Navier solution is applied to resolve the stresses and displacements due to uniformly distributed load imposed on thin, homogenous, simply supported rectangular plate. This plate theory is an extension of the classical Kirchhoff–Love plate theory, and is applicable to steel plates with length-to-thickness ratio of 50–100; the modelled steel plate specimen with rectangular holes was modelled accordingly. The governing equations derived from the Navier solution obtained from Reddy [16] are presented in Equations (1) and (2).

The Navier solution equation for displacement is expressed as such:

(1) $$w_{o}\left(x,y\right)=\frac{16q_{0}}{\pi^{6}D}\overset{\infty }{\underset{m=1}{\sum }}\overset{\infty }{\underset{n=1}{\sum }}\frac{sin\frac{m\pi x}{a}sin\frac{n\pi y}{b}}{mn{\left(\frac{m^{2}}{a^{2}}+\frac{n^{2}}{b^{2}}\right)}^{2}}\;m=1,3,5,\ldots \mathrm{and}\;n=1,3,5,\ldots$$

where *a* and *b* correspond to the plate’s length and width, while *D* denotes the flexural rigidity of the plate. The Navier solution equation for stress is:

(2) $$q\left(x,y\right)=\sum_{m=1}^{\infty }\sum_{n=1}^{\infty }\frac{16q_{o}}{\pi^{2}}sin\frac{m\pi x}{a}sin\frac{n\pi y}{b}\;m=1,3,5,\ldots and\;n=1,3,5,\ldots$$

Another analytical method that can be used to calculate and predict the stresses and displacements of a simply supported rectangular plate under uniform distributed loading is the Roark’s Formulas. Assuming that the plate is flat with straight boundary edges and uniform thickness, the maximum stress and displacement at the center of the plate can be estimated using Young and Budynas [17] formula shown in Equations (3) and (4).

(3) $$\sigma_{max}=\sigma_{b}=\frac{\beta qb^{2}}{t^{2}}$$

(4) $$y_{max}=\frac{-\alpha qb^{2}}{Et^{3}}$$

where *E* is the modulus of elasticity of the plate and *t* is the plate thickness. The values of α and ß used in Equations (3) and (4) are based on values recommended by Young and Budynas [17].

In this study, the Navier solution and Roark’s Formulas serve as benchmarks for comparison and results validation with the ANSYS simulation outcomes.

## 3. Methodology

### 3.1. Modelling of Steel Plates with Rectangular Openings

**Figure 2:**
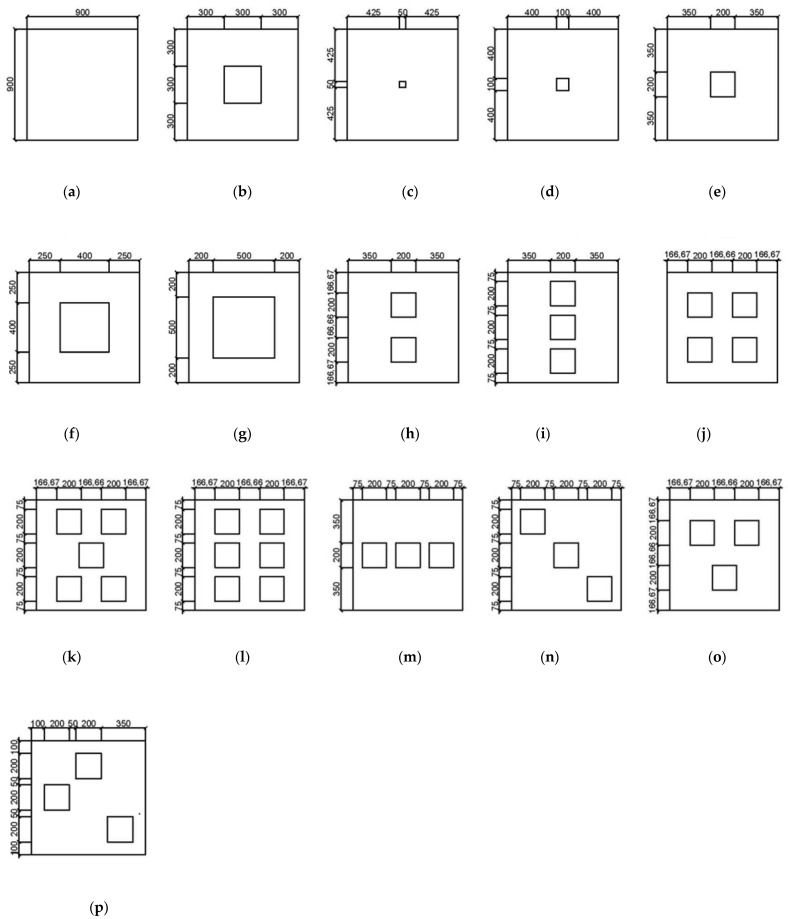
Visual schematics of the modelled specimens. (**a**) S1; (**b**) S2, S3, S4, S5 & S6; (**c**) S7; (**d**) S8; (**e**) S9; (**f**) S10; (**g**) S11; (**h**) S12; (**i**) S13; (**j**) S14; (**k**) S15; (**l**) S16; (**m**) S17; (**n**) S18; (**o**) S19; (**p**) S20.

The modelling of steel plates with rectangular holes was performed using ANSYS software. The process of modelling consisted of three main stages, namely pre-processing, solution and post processing. In the pre-processing phase, the geometry of the plates was modelled, so that each one had different parameters to test their effect on the ultimate strength of the steel plates. The modelled plates have different opening aspect ratio, plate thickness, opening positioning and the boundary conditions. Detailed parameters and visual geometry of the modelled specimens are shown in Table 1 and Figure 2, respectively.

The steel plate specimens with rectangular holes have a fixed plate dimension of 900 mm × 900 mm, and their material properties are set as structural steel. The steel plate specimens were further grouped according to the design parameters with potential influence on the ultimate strength of steel plates. The plate thicknesses of specimens S-2, S-3, S-4, S-5 and S-6 were set to 18 mm, 20 mm, 22 mm, 24 mm, and 26 mm, respectively, to investigate the effects of plate thickness on the ultimate strength of steel plates having rectangular openings. Additionally, specimens S-3, S-7, S-8, S-9, S-10 and S-11 were modelled using different opening aspect ratios in order to quantify the influence of opening aspect ration on the ultimate strength. Other than that, the results from plate specimens S-9, S-12, S-13, S-14, S-15 and S-16 were used to investigate the influence of opening numbers on the ultimate strength. On the other hand, the outcomes of specimens S-13, S-17, S-18, S-19 and S-20 were compared in order to evaluate the effect of opening positioning on plate performance. Lastly, the S-3 plate specimens with varying boundary conditions were tested to determine the effects of boundary conditions on the ultimate strength of steel plates with rectangular holes. The detailed groupings of all plate specimens are shown in Table 2.

### 3.2. Static Analysis of Steel Plates with Rectangular Holes—FEA

**Figure 3:**
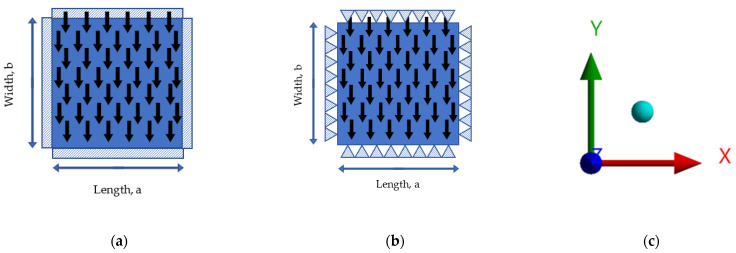
Boundary conditions of numerical models. (**a**) Plate with fixed boundary conditions; (**b**) plate with simply supported boundary conditions; (**c**) coordinate system of plates (x,y,z).

In ANSYS software, plate specimens are normally modelled as shell elements of type Shell181 or Shell281. These types of shells are suitable for the modelling of rectangular plates, and thus, for this project, the former shell type was chosen for the simulation. The plate’s shells were then meshed into smaller and simpler shell elements, where the smaller the meshed shell elements, the better the accuracy of the model solution, although this increases the computation time and the associated costs. Therefore, a suitable mesh size must be selected to provide accurate outcomes with reasonable computing time and cost. The plate specimens were modelled as structural steels, with Young’s modulus of 200 GPa and Poisson’s ratio of 0.3. Except for the plate specimens in group V, all specimens were simply supported, with a uniform pressure of 0.1 MPa acting along the plate specimen’s *z*-axis direction, as shown in Figure 3. Results and discussions are presented in the following section.

## 4. Results and Discussion

### 4.1. Comparison and Validation of FEA Results with Analytical Methods

**Figure 4:**
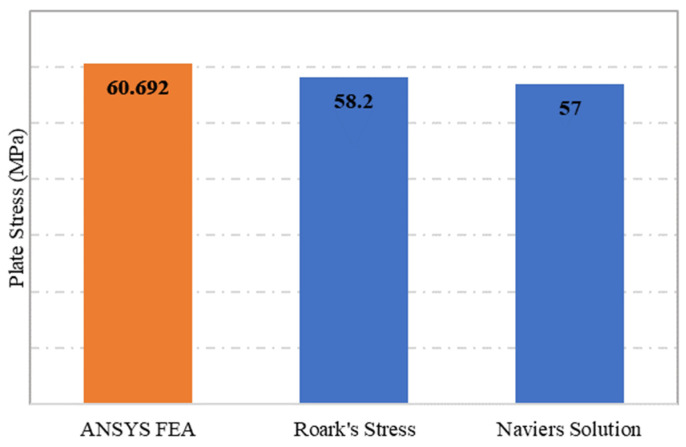
Plate maximum stress comparison between ANSYS FEA, Roark’s stress and Navier solution.

The plate specimen S-1, which was modelled as unperforated steel plate, was subjected to a static structural simulation under the SHELL181 programme. The simply supported plate S-1 was subjected to a pressure load of 0.1 MPa, which resulted in a maximum stress of 60.92 MPa. From the two analytical methods adopted for calculating the stress of unperforated rectangular panels, the Navier solution’s stress equation calculated the stress at 57 MPa, while from the stress equation of Roark’s Formulas, the maximum stress value determined was 58.2 MPa, as shown in Figure 4. This indicates that the ANSYS simulation results were 7% higher than the results obtained from the two analytical methods. This indicates that the results from ANSYS FEA, Navier solution and Roark’s Formulas are in good agreement.

**Figure 5:**
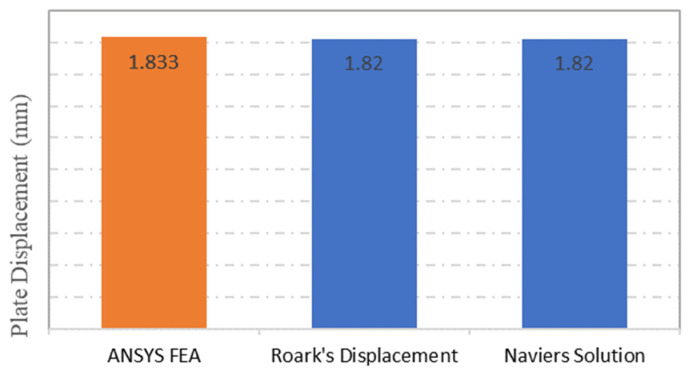
Plate maximum displacement comparison between ANSYS FEA, Roark’s Stress and Navier solution.

The same observation can be seen in the displacement results obtained from ANSYS numerical simulation, Roark’s Formulas and the Navier Solution, which were recorded as 1.83 mm for ANSYS simulation and 1.82 mm for both of the analytical methods respectively, as depicted in Figure 5. The comparison between the displacement results shows a good agreement, as the percentage of error was limited to 1% only.

**Figure 6:**
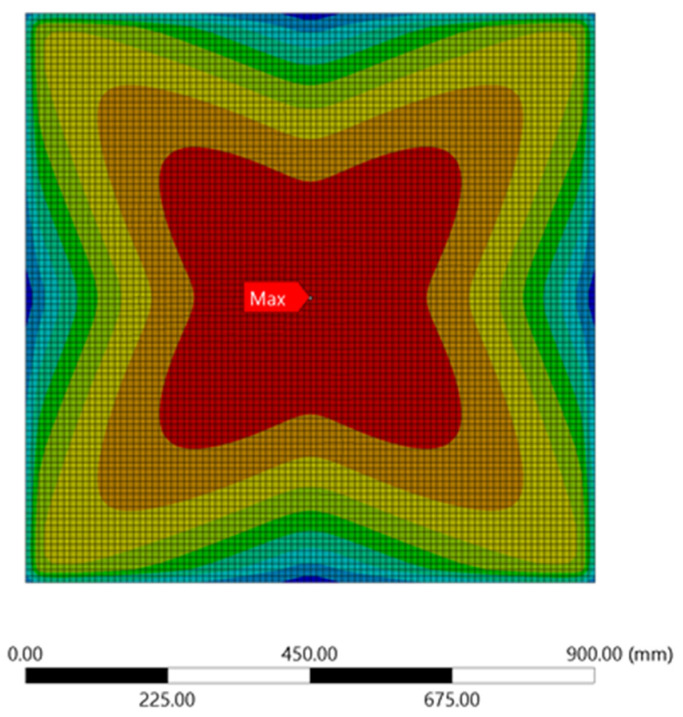
Stress contour for the unperforated steel plate.

**Figure 7:**
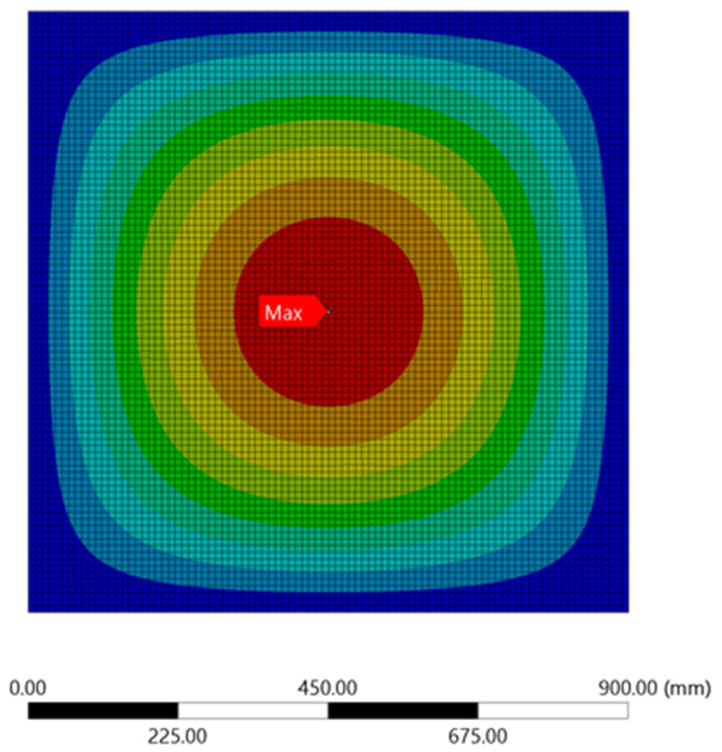
Displacement contour for the unperforated steel plate.

Further, observation on the stress and displacement of ANSYS simulation results revealed that the maximum stress and displacement resulting from the applied load of 0.1 MPa were both observed at the centre of the plate, as shown in Figure 6 and Figure 7, respectively. Moreover, the maximum stress and displacement sustained by the unperforated steel plate specimens occurred mainly on the tensioning face of the steel plates. This plate contour information can be used by designers to investigate the stress of steel plate specimens and the corresponding displacement concentration, which will help to draw appropriate conclusions during the comparison of varying steel plates with rectangular holes [2] under different configurations and design parameters.

With the comparison and validation of stress and displacement results of unperforated steel plates attained from the ANSYS FEA simulation, Navier Solution and Roark’s Formulas, one can observe that the analytical methods showed a satisfactory agreement with the FEA simulation. This indicates that ANSYS FEA can be used to accurately determine the stresses and displacements of steel plates with rectangular holes, as discussed in the following sections.

### 4.2. Influence of Plate Thickness

**Figure 8:**
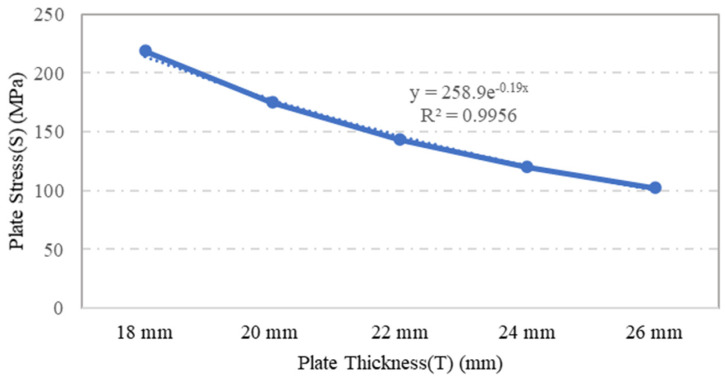
Influence of plate thickness on the plate maximum stress.

**Figure 9:**
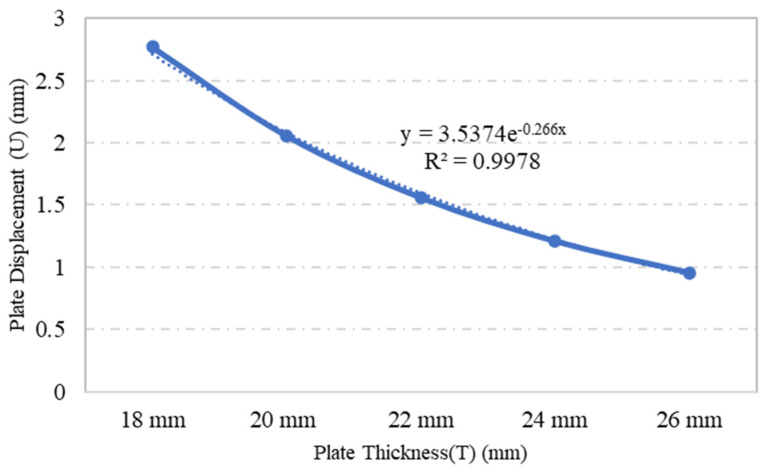
Influence of plate thickness on plate maximum displacement.

Plate specimens S-2, S-3, S-4, S-5 and S-6, which were modelled using the same plate dimensions and opening geometry but with varying plate thicknesses, were assessed in terms of their stress and displacement under a loading of 0.1 MPa through ANSYS numerical simulation. The variations in plate thickness had a significant impact on the stress and displacement. The outcome of the simulation indicated that as the plate thickness increased, the stress and displacement of the plate decreased, as shown in Figure 8 and Figure 9, respectively. For example, when the plate thickness was increased by 44%, plate stress was reduced by 53%. Moreover, the exponential trendline of R^2^ = 0.995 in Figure 8 illustrates a decrease in plate’s stress values as the plate thickness increases. This inversely proportional relationship was caused by the increase in the rigidity and stiffness of plate, which escalated the overall stress capacity of the plate as the plate thickness increased.

**Figure 10:**
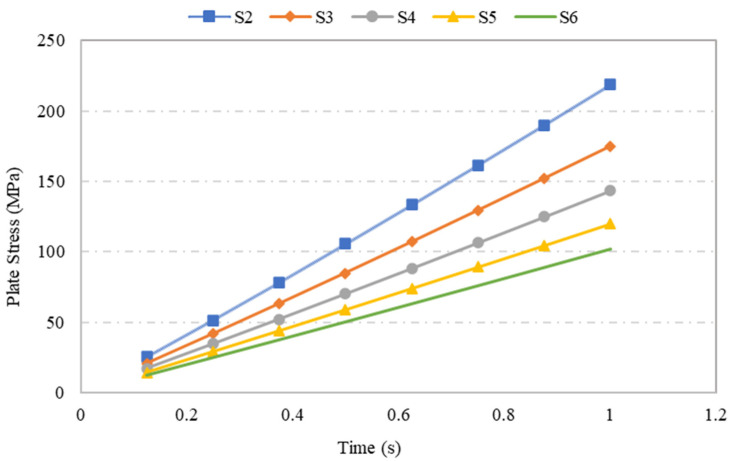
Variation in maximum stress of steel plate specimens with varied plate thickness over time.

Comparison between the lowest and the highest values of maximum stress and displacement showed a reduction of 114% in stress and a 190% reduction in displacement. Further, looking into the variation in steel plate specimen maximum stress values with respect to time, one can observe that the maximum stress increased with time under an applied load of 0.1 MPa. Further, as presented in Figure 10, the comparison of diverse steel plate specimens with different plate thicknesses indicated that there was an indirectly proportional relationship between the plate thickness and the maximum stress observed, although in all the cases, the ANSYS simulation produced maximum stresses that did not exceed the yield stress of 250 MPa.

**Figure 11:**
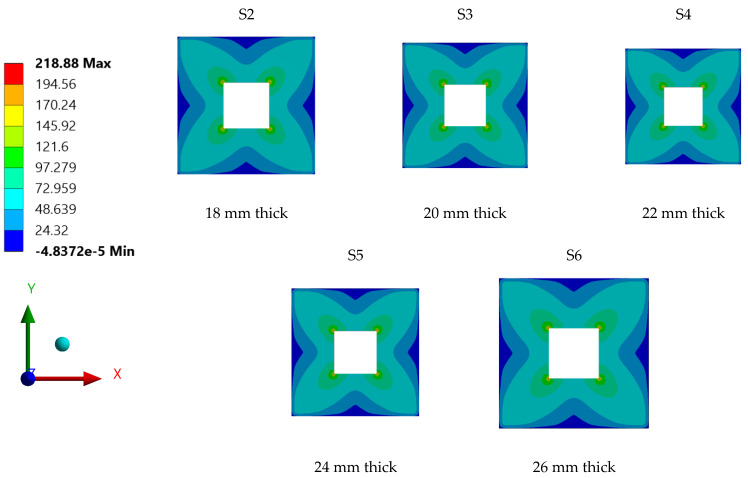
Stress distribution contours for individual steel plate specimens with varied wall thickness.

Further, Figure 11 shows the stress distribution contours for individual steel plate specimens with varied plate thickness. Generally, all steel plates had similar stress contour distribution patterns; this is attributed to the plates’ geometry, as all tested samples had typical geometry, regardless of their wall thickness. Another common characteristic the specimens shared was the maximum stress of the plate, which was mainly concentrated at the corners of openings, due to stress concertation effects.

### 4.3. Influence of Plate Opening Aspect Ratio

**Figure 12:**
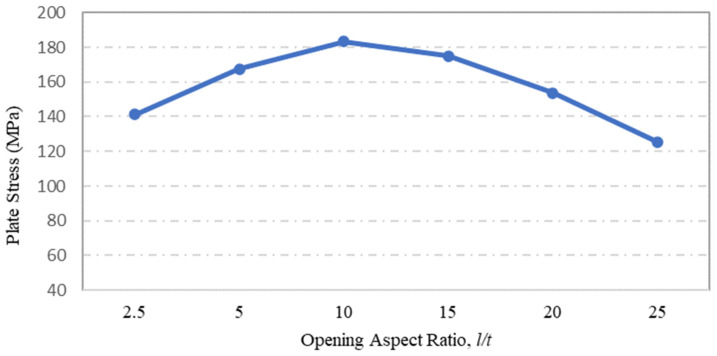
Influence of plate opening aspect ratio on maximum stress.

**Figure 13:**
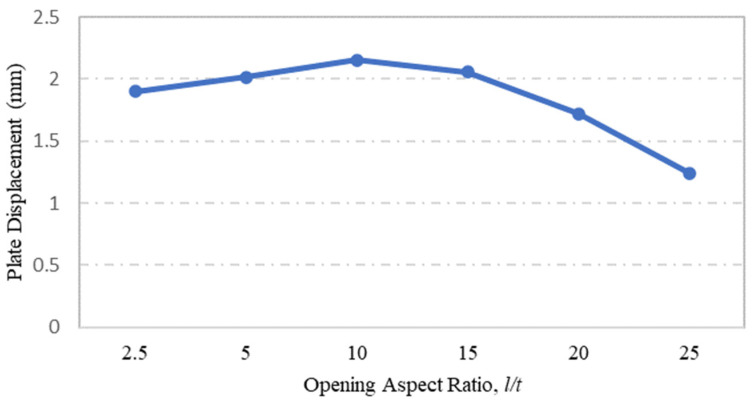
Influence of plate opening aspect ratio on maximum displacement.

Steel plate specimens modelled to investigate the influence of plate opening aspect ratio on the strength capacity of steel plates with rectangular holes were S-7, S-8, S-9, S-3, S-10 and S-11, and their corresponding stress and displacement values are shown in Figure 12 and Figure 13, respectively. These steel plate specimens had the same plate dimensions and thickness, but their opening aspect ratios were the basis of comparison. From the FEA simulation results presented in Figure 12, one can observe that the maximum stress on the plate was significantly influenced by the opening aspect ratio. The maximum stress increased with the aspect ratio to reach its maximum value at *l/t* =10, prior to decrease gradually as the aspect ratio exceeded *l/t* = 10. The same result can be seen for the displacement of steel plates with regard to the plate opening aspect ratio, as shown in Figure 13.

**Figure 14:**
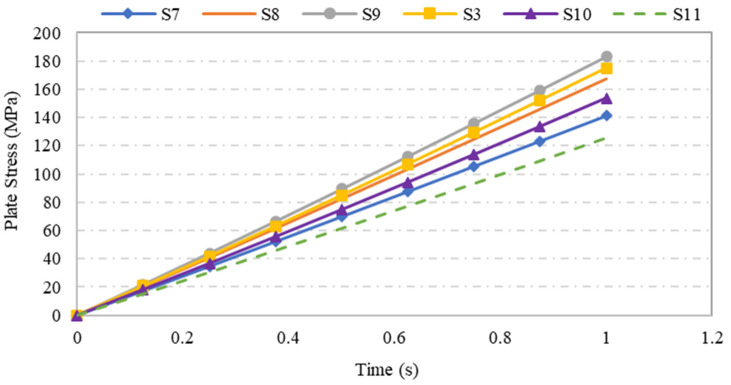
Variation in maximum stress of steel plate specimens with varying opening aspect ratios over time.

Figure 14 shows the time series records for the variation in maximum stress on steel plates as a function of opening aspect ratio. From the graph, one can observe a linear relationship between the maximum stress on steel plate specimens and the duration of test for the different specimens. Further, one can observe that S-9 plate specimen with aspect ratio of *l/t* = 10 has the highest maximum stress, while the plate specimens with aspect ratios greater than *l/t* = 10 experienced a lower stress value.

**Figure 15:**
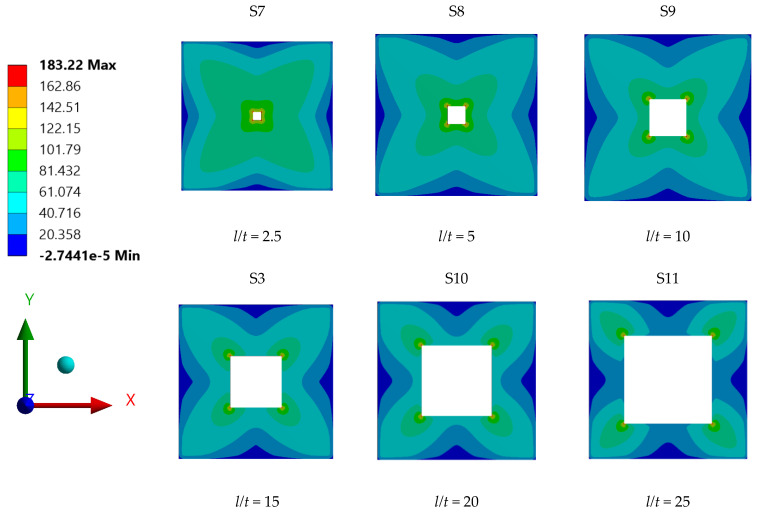
Stress distribution contours for individual steel plates with varying opening aspect ratios.

The stress contour plots for the various steel plate specimens shown in Figure 15 were further examined to better understand and interpret the corelation between the plate opening aspect ratio and the maximum stress. It can be observed that the plates with opening aspect ratio not exceeding *l/t* = 10 has high stress concentrations, mainly distributed around the opening corners and edges, which indicates that the applied pressure of 0.1 MPa was transmitted and transferred to the supports by the edges of plate openings’ corners. On the other hand, for plate specimens with opening aspect ratios higher than *l/t* = 10, the high stress concentrations were observed to be carried by the plate openings’ corners only. The transfer of the plate stress occurred mainly from the corners of the openings to the supports; this was seen as an ineffective stress transfer, as the whole plate surface was not fully utilised. Accordingly, the reduction in maximum stress for steel plates with aspect ratios over *l/t* = 10 was expected, due to the possibility of failure mode occurring at the opening corners. This could be associated with the presence of a tension tear at the corners of plate, which would cause the maximum stress and displacement of the plate to drop. According to Zhao et al. [10] investigation report on shear stress of steel plates with openings through experimental method and numerical analysis, one steel plate specimen exhibited a failure mode known as tension tear, which occurred at the corners of the plate openings. Upon validating the shear capacity of these particular plates with the shear stress value obtained from ANSYS FEA, the shear stress yielded by the two methods showed good agreement. The stress concentration was also seen to be highest at the corners of the openings of said steel plate specimen.

### 4.4. Influence of Plate Opening Numbers

**Figure 16:**
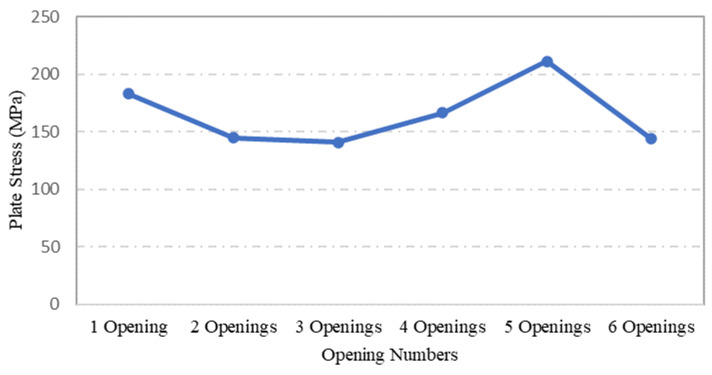
Influence of plate opening numbers on maximum stress.

An evaluation was conducted to determine the influence of opening numbers on the performance of steel plates, using specimens S-9, S-12, S-13, S-14, S-15 and S-16, in which the plate geometry and openings size remained the same, while the number of openings was varied. The effect of openings on stress and displacement was evaluated using ANSYS FEA simulations. Figure 16 shows that plate specimen S-9, with one centric opening, and specimen S-15, with 5 openings, experienced the highest maximum stress values. The similarity between the two plates is that both plates consists of oneopening of equal size at the centre of the plate. This could be the reason for the jump in their maximum stress values.

**Figure 17:**
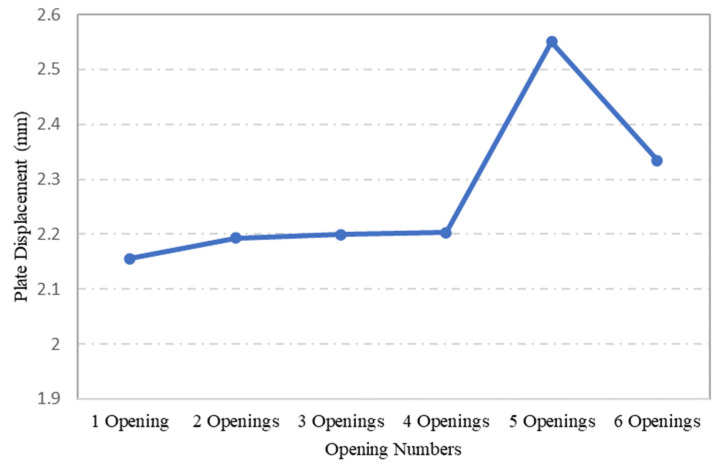
Influence of opening numbers on the maximum displacement of plates.

As for the maximum displacements of steel plates with varying opening numbers through ANSYS FEA, as shown in Figure 17, the highest displacement recorded was for plate specimen S-15, which had 5 openings. However, further comparison between the stress and displacement values of the steel plates showed that these parameters did not follow the same pattern. Particularly, plate specimen S-9 maximum stress under the applied load was the second highest, while in terms of displacement, the attained value was the lowest. This might be attributed to its one centric opening, which left the plate specimen S-9 with more surface area than the remaining plate specimens. However, in the case of plate specimen S-13, which also had an opening at its centre, the maximum stress developed was the lowest. This is due to the arrangement of openings and their number, arranged as three openings in a linear pattern passing along the centre line of the plates. This arrangement apparently redistributed the stress concentration factor at the corner of openings.

**Figure 18:**
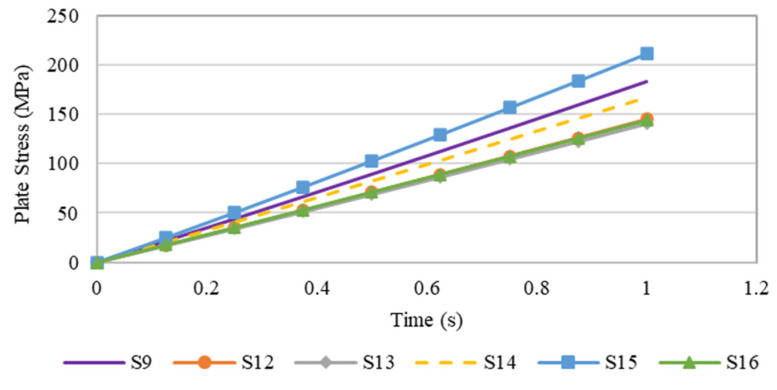
Graph plot of the maximum stress of steel plate specimens with different plate opening numbers over time.

Figure 18 shows the variation in maximum stress for steel plates with different openings as a function of time. As highlighted earlier in the previous section, plate specimen S-15 had the highest maximum stress, followed by plate S-9. Generally, the variation in stress over time was linearly proportional, although the maximum stress attained by each plate with various opening did not exceed the yield strength of steel.

**Figure 19:**
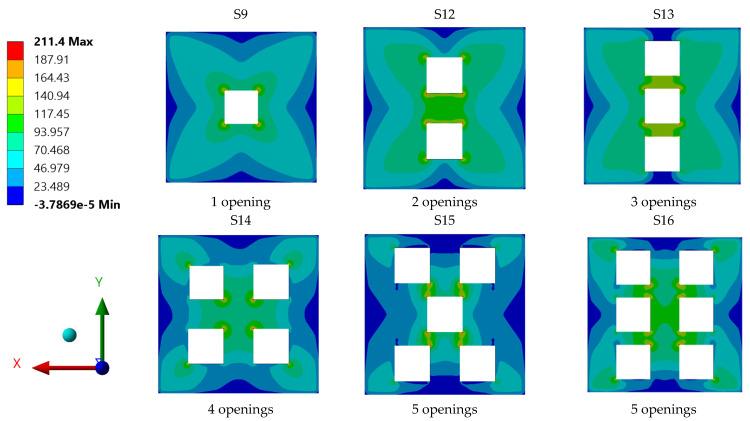
Stress distribution contours for individual steel plates with different plate opening numbers.

In view of the stress distribution contours of plates with various openings presented in Figure 19, one can observe that all plate specimens had diverse stress concentration contours. Close observation of the stress contours suggests that plate specimens S-15 and S-9 experienced the highest stress values. These high stress values were mainly concentrated around the corners of the plate openings. As for the other steel plate specimens, their maximum stress values were comparatively low; this can be attributed to the efficient transmission of the applied stress through their whole surface areas to the support edges and corners.

### 4.5. Influence of Plate Opening Position

**Figure 20:**
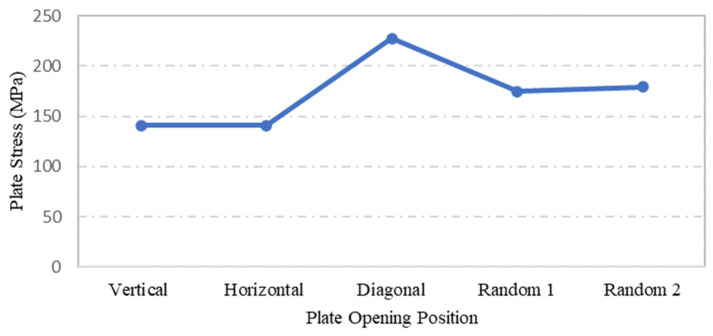
Influence of plate opening positions on plate maximum stress.

**Figure 21:**
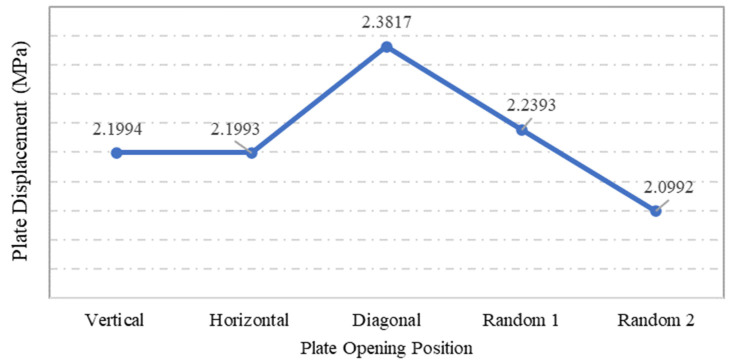
Influence of plate opening positions on plate maximum displacement.

Steel plate specimens S-13, S-17, S-18, S-1, and S-20 were modelled with the same plate geometry, thickness, opening aspect ratio and opening numbers. However, the distinct feature of each was the position and configuration of openings. The effects of these openings on strength capacity and displacement of plates under the applied loadings were investigated. The solutions of ANSYS FEA on the abovementioned steel plates with differing opening positions are presented in Table 3. One of the main observation from the graphs shown in Figure 20 and Figure 21 is that specimens S-13 and S-17 have identical values for the maximum stress and displacement. The other remarkable observation is the maximum stress and displacement of plate S-18, which consisted of diagonal openings. Regarding the former observation, a reasonable explanation for the plate specimen S-13 and S-17 having equal stress and displacement values could be their square geometry, their identical properties, and their opening aspect ratios. The plate specimens S-13 and S-17 were similar in terms of dimension, except that the opening configuration was different, and theoretically, it was expected that these two plate specimens would result in identical results. With regard to the latter observation, the fact that plate specimen S-18 was characterised with the highest stress and displacement outputs can be associated with the positioning of the openings, which were placed on the critical path of stress distribution. Thus, the presence of diagonal openings caused disturbance to the strength capacity and consequently amplified the stress distribution and the stress concentration of the plate.

**Figure 22:**
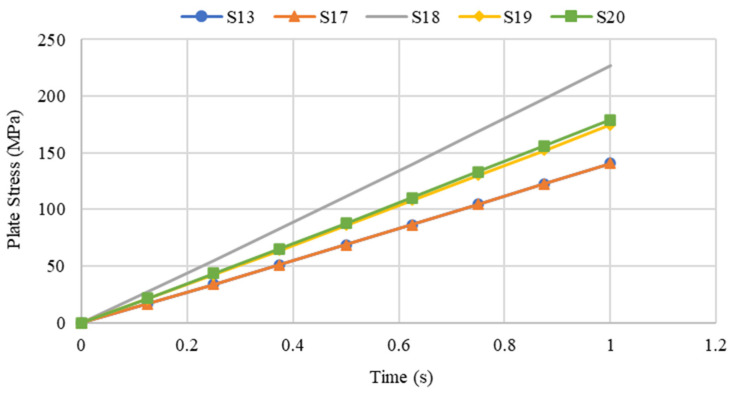
Variation in maximum stress of steel plate specimens with differing plate opening positions.

The highest maximum stress for all the plate specimens occurred at the time where the applied pressure was at its peak of 0.1 MPa. The plate specimen S-18, yielded the highest maximum stress also achieved the largest maximum stress results over the time interval, as shown in Figure 22.

**Figure 23:**
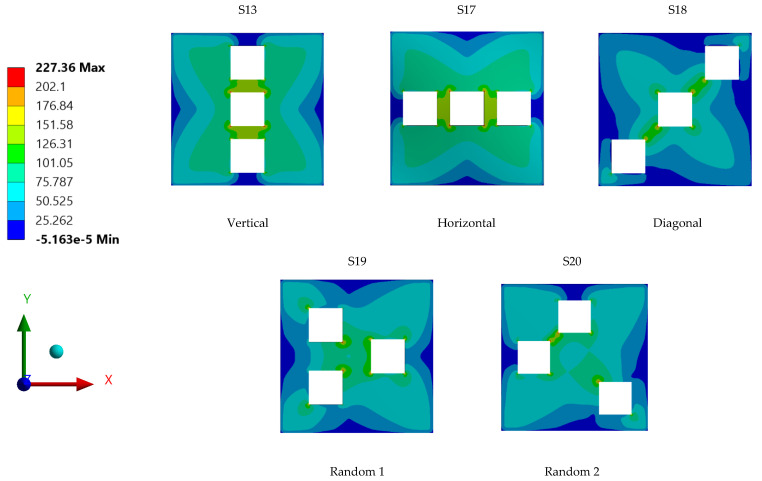
Stress contour areas for individual steel plate specimens with differing plate opening positions.

Meticulous analysis and evaluation of the stress contours of the steel plate specimens with differing opening positions and configurations, as displayed in Figure 23, show that every plate specimen had its own unique stress distribution contour portraying the stress distribution of the applied load throughout the plate surface. Plate specimens S-13 and S-17 had common stress and displacement values, but their stress distribution contour areas differed from one another. 

As for plate specimen S-18, which attained the highest stress and displacement among the different specimens, the position of opening on the plate was found to interdict the distribution of stress experienced by the plate. The maximum stress concentration can be observed at the coerners of openings, which lay along the tensioning plane of the plate specimen. The positions of the openings aligned with the stress distribution path of the plate specimen may be the reason behind the high stress and displacement of the steel plate specimen S-18 with diagonal opening structures. Further, in the analysis of the result shown in Figure 23, it can be seen that this plate had the lowest maximum displacement. This might be due to the absence of any openings near the plate’s centre, in contrast with the other plates.

### 4.6. Influence of Plate Boundary Condition

**Figure 24:**
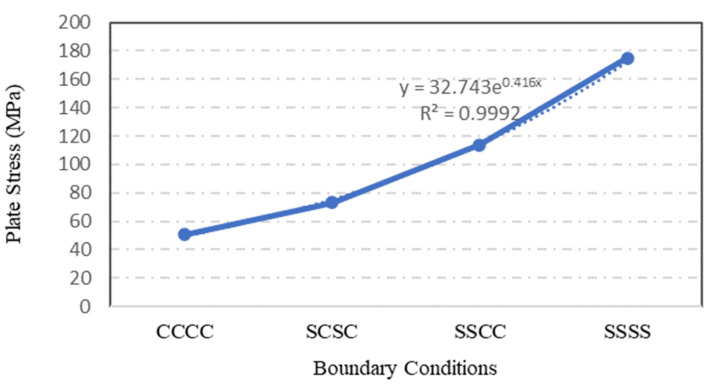
Variation in plate maximum stress with different boundary conditions.

The types of boundary conditions constraining the plate edges were assessed to quantify their influence on the plate strength and capacity. Four identical S-3 plates with different boundary conditions were simulated statically using the ANSYS software. Although only two types of boundary condition were applied to the steel plate specimens, which are simply supported with clamped boundary conditions, the configuration of boundary conditions at each individual plate was varied. In Figure 24, the symbol ‘C’ indicates a clamped plate’s edge while the symbol ‘S’ implies that the plate’s edge -is simply supported. Therefore, a CCCC boundary condition configuration means that the steel plate was clamped on all its four edges.

**Figure 25:**
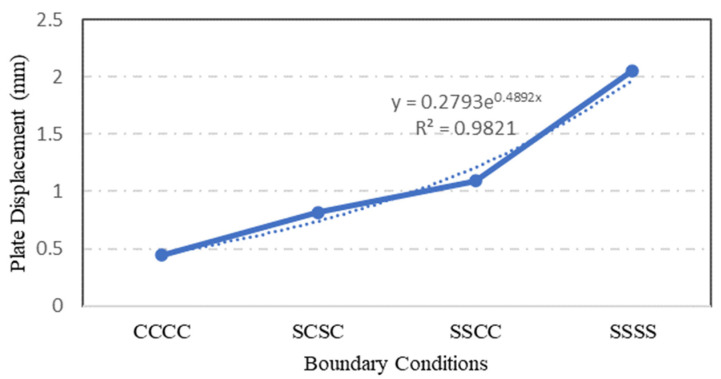
Variation in maximum displacement with different boundary conditions.

The type of boundary condition applied to the plate specimen’s edges determined the degree of freedom at the edge of steel plate. From the ANSYS simulation of the four S-3 plates with different boundary conditions, it was found that the plate specimen with CCCC boundary conditions has the lowest stress value, followed by SCSC, SSCC and SSSS plates. As depicted in Figure 24 and Figure 25, the stress and displacement varied with the degree of freedom of plate specimens. Plate specimen supports with freedom to rotate have experienced higher stresses and displacements.

**Figure 26:**
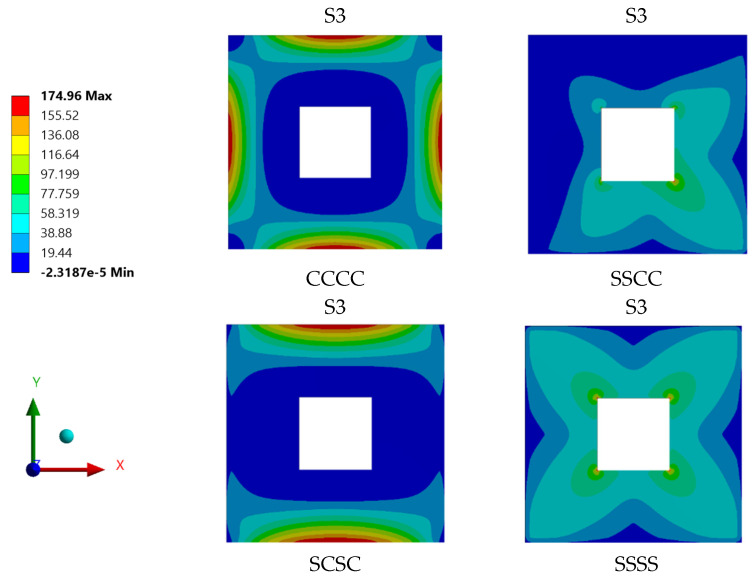
Stress distribution for individual plate specimens with different boundary conditions.

The stress contour plot for plate specimens under the influence of varying boundary conditions is presented in Figure 26. In the case of plate specimens with fixed supports, the applied pressure was distributed and carried out by the supports and plate edges. The maximum stress was concentrated at the edges of the plate near the fixed support. Meanwhile, for plate specimens with simply supported edges, their stress distribution was observed to be resisted by the plate specimen itself, with the maximum stress concentration occurred at the corners of the plate’s openings.

Generally, plate specimens with CCCC boundary conditions experienced the lowest maximum stress. On the other hand, the plate specimens with SSSS boundary conditions resisted the applied pressure and transferred the stress throughout the plate surface to the supports.

### 4.7. Parameter-Optimised Steel Plate with Rectangular Holes

As discussed earlier with regard to the influence of plate thickness on the stress and displacement values of steel plates with rectangular holes, the larger the plate thickness, the higher the strength capacity of the steel plate with rectangular holes in withstanding the applied loads. Increasing the plate thickness increases the rigidity and stiffness of the steel plate with rectangular holes. The plate thickness for the optimised steel plate with rectangular holes was 26 mm.

As for the opening aspect ratio for the optimised steel plate with rectangular holes, the smaller the opening aspect ratio, the better its stress capacity. A small opening on the steel plate means that the steel plate has more material and area for distribution and transmission of the stresses. 

Further, the number of openings on the steel plate must be minimal, as the number of openings on the steel plate can interfere with the transmission of stress from the centre plate to the plate supports. The positioning of openings is also crucial in ensuring the stress and displacement capacity of the steel plate with rectangular holes. 

Based on the FEA results, the boundary condition chosen for the optimised steel plate with rectangular hole was the simply supported condition on all four sides of the plate. The intention of these optimisations was to ascertain the capacity and performance of the optimised steel plate with rectangular hole in resisting the applied load and analysing the stress and displacement occurring under the pressure loadings. The optimum design parameters based on FEA result are presented in Table 4.

Performing ANSYS FEA on the steel plate with rectangular holes clearly showed that stress and displacement outputs were sensitive to the parameters investigated. The optimised steel plate with rectangular hole experienced a maximum stress and displacement of 84.107 MPa and 0.88283 mm, respectively, as shown in Table 5. 

## 5. Conclusions

A parametric study was conducted to investigate the influence of selected parameters on the ultimate capacity of steel plates with rectangular openings subjected to axial stress. The steel plates with openings were modelled using non-linear FEA software: ANSYS. The results obtained from ANSYS simulation for the stress and displacement of steel plates without openings were validated using the Navier Solution and Roark’s Formulas. ANSYS simulation showed good agreement with the results obtained from the two analytical methods for the steel plates without openings. The influence of plate thickness, opening ratio, opening number, opening position and boundary conditions on steel stress and displacement were investigated. The following conclusions were drawn based on the parametric study:

- Maximum stress and displacement decreased exponentially with increasing thickness of the steel plates with rectangular openings. The exponentially decreasing trendlines are presented in equations with good regression value of R^2^ = 0.99. The comparison of maximum stress between the lowest and the highest values show a 114% reduction in the maximum stress and 190% reduction in the displacement values.
- The maximum stress and displacement decreased when the aspect ratio was more than 10. This finding is helpful for designers in selecting suitable dimensions for openings at the preliminary design stage. However,–once an opening with a larger aspect ratio is introduced, the plate may fail due to tension tears at the corners of openings.
- Increasing the number of steel plate opening led to an increase in the maximum stress and displacement, but the steel plate specimens with a opening at the centre experienced higher stress values, due to the inefficient stress distribution throughout the steel plates.. Plate specimen S-13 was an outlier in this case, as it had an opening at its centre, it experienced the lowest maximum stress, which represents a 50% reduction compared to plate specimen S-15.
- The position and configuration of the plate openings were more critical when the openings were placed along the stress transmission path, which could interdict with the stress distribution and cause high stress concentration points at the opening corners. The analysis showed that vertical, horizontal and random openings positions yielded lower displacement values as compared to others plates. Generally, a 61% jump in maximum stress was observed in the steel plate with diagonal opening arrangement compared with the plate specimen achieving lowest stress.
- The type of boundary conditions applied to the steel plate specimens determined the degree of freedom given to the plate specimens. Generally, plates with a higher degree of freedom experienced higher stress and larger displacements. Plate specimens with all sides clamped shown a 245% reduction in stress when compared to the plate specimens having simply supported edges.

## Figures and Tables

**Table 1:** Detailed parameters of the modelled specimens.

Specimen No.	Opening Shape	Opening Dimensions	Plate Thickness	Opening Number
S-1	-	-	20.0	-
S-2	Rectangular	300 × 300	18.0	1
S-3	Rectangular	300 × 300	20.0	1
S-4	Rectangular	300 × 300	22.0	1
S-5	Rectangular	300 × 300	24.0	1
S-6	Rectangular	300 × 300	26.0	1
S-7	Rectangular	50 × 50	20.0	1
S-8	Rectangular	100 × 100	20.0	1
S-9	Rectangular	200 × 200	20.0	1
S-10	Rectangular	400 × 400	20.0	1
S-11	Rectangular	500 × 500	20.0	1
S-12	Rectangular	200 × 200	20.0	2
S-13	Rectangular	200 × 200	20.0	3
S-14	Rectangular	200 × 200	20.0	4
S-15	Rectangular	200 × 200	20.0	5
S-16	Rectangular	200 × 200	20.0	6
S-17	Rectangular	200 × 200	20.0	3
S-18	Rectangular	200 × 200	20.0	3
S-19	Rectangular	200 × 200	20.0	3
S-20	Rectangular	200 × 200	20.0	3

**Table 2:** Assigned groupings of the modelled specimens.

Group Number	Specimen	Research Objectives
Group I	S-2, S-3, S-4, S-5, and S-6	Influence of plate thickness on ultimate strength
Group II	S-3, S-7, S-8, S-9, S-10, and S-11	Influence of opening aspect ratio on ultimate strength
Group III	S-9, S-12, S-13, S-14, S-15, and S-16	Influence of opening numbers on ultimate strength
Group IV	S-13, S-17, S-18, S-19, and S-20	Influence of opening position on ultimate strength
Group V	S-3 (All edges simply supported), S-3 (All edges clamped), S-3 (CSCS) and S-3 (SSCC)	Influence of boundary conditions on ultimate strength

**Table 3:** Steel plate specimens with differing opening positions and their respective maximum stress and displacement investigated by ANSYS.

Plate Specimen	Opening Position	Max Stress (MPa)	Max Displacement
S13	Vertical	140.8	2.1994
S17	Horizontal	140.8	2.1993
S18	Diagonal	227.36	2.3817
S19	Random 1	174.76	2.2393
S20	Random 2	179.26	2.0992

**Table 4:** Summary of optimised parameters for the modelling of the steel plate with rectangular holes.

Plate Parameters	Optimum Values
Geometry	900 × 900 mm
Thickness	26 mm
Opening Aspect Ratio	50 × 50 mm
Number of Opening	1 Openings
Opening Position	Plate Centre
Boundary Conditions	Simply Supported

**Table 5:** Maximum stresses and displacement values for the optimised steel plate with rectangular hole with respect to time.

		Optimized Steel Plate with Rectangular Hole	
Time	Applied Pressure (MPa)	Max Stress (MPa)	Max Displacement
0	0	0	0
0.125	0.0125	10.389	0.11053
0.25	0.025	20.818	0.22104
0.375	0.0375	31.283	0.33152
0.5	0.05	41.784	0.44195
0.625	0.0625	52.319	0.55231
0.75	0.075	62.886	0.66259
0.875	0.0875	73.482	0.77276
1	0.1	84.107	0.88283

## Data Availability

Not applicable.
